# Protein Homology Network Families Reveal Step-Wise Diversification of Type III and Type IV Secretion Systems

**DOI:** 10.1371/journal.pcbi.0020173

**Published:** 2006-12-01

**Authors:** Duccio Medini, Antonello Covacci, Claudio Donati

**Affiliations:** Cellular Microbiology and BioInformatics Unit, Novartis Vaccines and Diagnostics, Siena, Italy; Institut für Molekulare Infektionsbiologie der Universität, Germany

## Abstract

From the analysis of 251 prokaryotic genomes stored in public databases, the 761,260 deduced proteins were used to reconstruct a complete set of bacterial proteic families. Using the new *Overlap* algorithm, we have partitioned the Protein Homology Network (PHN), where the proteins are the nodes and the links represent homology relationships. The algorithm identifies the densely connected regions of the PHN that define the families of homologous proteins, here called PHN-Families, recognizing the phylogenetic relationships embedded in the network. By direct comparison with a manually curated dataset, we assessed that this classification algorithm generates data of quality similar to a human expert. Then, we explored the network to identify families involved in the assembly of Type III and Type IV secretion systems (T3SS and T4SS). We noticed that, beside a core of conserved functions (eight proteins for T3SS, seven for T4SS), a variable set of accessory components is always present (one to nine for T3SS, one to five for T4SS). Each member of the core corresponds to a single PHN-Family, while accessory proteins are distributed among different pure families. The PHN-Family classification suggests that T3SS and T4SS have been assembled through a step-wise, discontinuous process, by complementing the conserved core with subgroups of nonconserved proteins. Such genetic modules, independently recruited and probably tuned on specific effectors, contribute to the functional specialization of these organelles to different microenvironments.

## Synopsis

Proteins evolved from a common ancestor are said to be homologues and to constitute a “family” with potentially similar structures, functions, and interactions. The problem of identifying “real” protein families based on amino acid sequence conservation has been the subject of extensive debate, because algorithms that search for pairwise homologies can miss important relations and produce false hits. The availability of a large number of sequenced genomes now allows us to map the full set of protein similarity relationships into a Protein Homology Network (PHN), and protein families appear naturally as dense, highly connected regions of the network. In this study, Medini, Covacci, and Donati describe a new method that identifies these regions of the PHN, and generate a set of protein families (PHN-Families) that correlate with protein function and phylogeny, with a quality comparable to family sets curated by human experts. The method is completely unsupervised and can be applied to any number of genomes. The authors test the biological relevance of the PHN-Families obtained by studying the members of Type III and Type IV secretion systems, showing that this classification can also be used to identify the evolutionary events that led to the formation of multiprotein structures.

## Introduction

The seminal observation of Margareth O. Dayhoff [1,2] that proteins evolved from a common ancestor into families [2,3] has been recognized as central in the speciation and diversification processes. In addition, the horizontal transmission of genetic material between different species is frequent, and has shaped the evolution of many living organisms [4,5], suggesting that the concept of the phylogenetic tree should be replaced by a phylogenetic network, where connections between different clades occur due to horizontal gene transfer [6]. These nontrivial inheritance patterns are more easily detectable once each gene product has been classified in a protein family, and correlated evolutionary history of different systems or system components become visible.

We have investigated the problem of reconstructing the evolutionary relationships amongst proteins and of classifying them into families from a topological point of view, by defining the Protein Homology Network (PHN). In the PHN, proteins are seen as nodes connected by links that represent the homology relations inferred by sequence similarity. In such a representation, protein families should appear as dense clusters disconnected from the rest of the network. Since alignment search algorithms can only approximate the real genetic distance between proteins, false or missing links alter the ideal structure of the network. Furthermore, proteins resulting from the fusion of two or more protein domains show homology with members of different families. As a consequence, regions of the network with a higher density of links are still recognizable, but family boundaries are more difficult to identify.

We have used the intrinsic transitivity of true homology relationships to define a new similarity measure among proteins, which allows us to identify unambiguously the densely connected regions of the network, which we define as PHN-Families. Based on this measure, we have devised an algorithm able to classify large sets of proteins into PHN-Families without human intervention.

To demonstrate the potential of this approach, we have studied the classification into PHN-Families of the structural components of two complex bacterial organelles, namely Type III [7–10] and Type IV [11–14] secretion systems (T3SS and T4SS, respectively), which are contact-dependent export systems widely spread among pathogenic and nonpathogenic bacteria. T3SS and T4SS are ancestrally related to other bacterial organelles, flagella [15,16], and the conjugative apparatus, respectively [17]. Both systems are frequently transferred horizontally between microorganisms, and some of their constituents form multidomain fusion proteins.

Experimental evidence and comparative analysis of both T3SS [7,16] and T4SS [11,13] have defined a set of characteristic functions that are conserved in the majority of known secretion systems. In both cases, the PHN-Families allow us to quickly identify a conserved core of proteins, shared with the ancestrally related apparatuses, which are present in more than 120 bacteria. At the same time we find that many other constituents, considered functionally homologous, are separated by a genetic divergence incompatible with a single family. An evolutionary profiling of both apparatuses, based on the PHN-Family classification of their members, suggests that T3SS and T4SS have been assembled through a step-wise, discontinuous process, by complementing the conserved core with distant genetic units. Such modules, often independently recruited, and probably tuned on specific effectors, determine the functional specialization of these organelles to different microenvironments.

## Results

**Figure 1:**
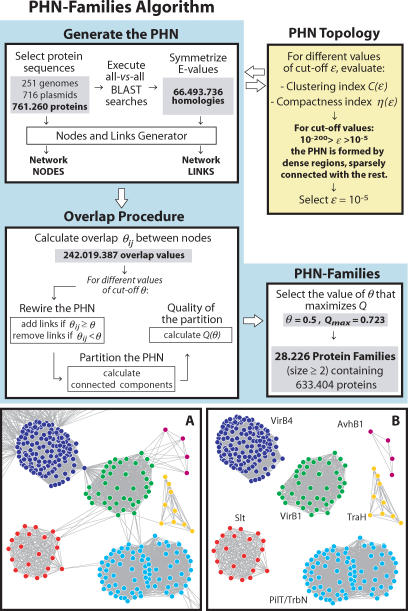
Overview of the Procedure (Top Panel) The four major steps used to define the PHN-Families are shown. The blue-shaded boxes (left and lower right) indicate the automated steps of the algorithm that, starting from a set of protein sequences, lead to the PHN-Families definition: i) generation of the network, ii) partitioning of the network for various cutoff values, iii) selection of the optimal cutoff. Values specific to the system analyzed in this study are shown on a gray background within the three boxes. The tan box (upper right) summarizes the investigation of the network topology. Since the PHN structure does not change upon addition of new sequences, this step does not need to be repeated when the sequence dataset is updated. (A,B) A graphical visualization ([31], see Protocol S1) of the PHN in proximity to the VirB1 proteins is shown. (A) shows the results before partitioning, and (B) shows the results after partitioning. Different colors indicate different PHN-Families. doi:10.1371/journal.pcbi. 0020173.g001

Results are organized as follows: in the first subsection we demonstrate that the PHN is formed by densely connected regions, which we identify as families of homologous proteins. In the second we describe the algorithm that identifies such families (see Figure 1), and we assess their quality. In the third subsection we analyze the PHN-Families classification of the T3SS and T4SS structural components, and finally we use PHN-Families to suggest a model for the diversification of the secretory apparatuses.

### The Protein Homology Network

**Figure 2:**
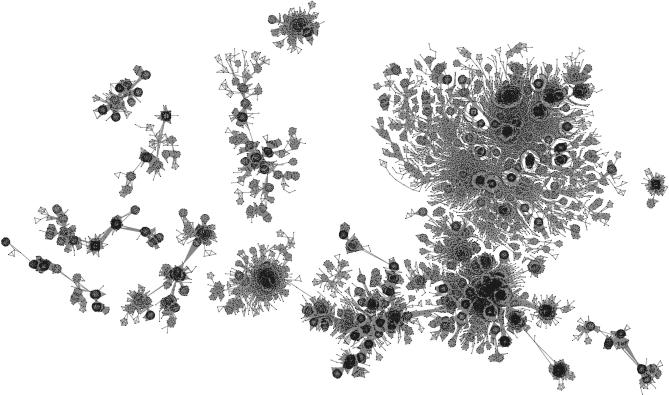
The Protein Homology Network A representation of a few of the largest connected components of the PHN for *ɛ* = 10^−30^ (39,321 nodes, 4.4 × 10^6^ links, see Protocol S1). Points represent proteins, and links represent sequence homology relationships with a BLAST E-value smaller than *ɛ*. The modular structure of the PHN is clearly visible in the figure, where many tightly connected groups of proteins appear to be linked together to form globally sparse connected components. By increasing the value of *ɛ,* the number of links wiring the network grows, causing many smaller components to coalesce into a single giant cluster. Moreover, many of the sparse points of the figure, which appear not to belong to any compact cluster, also join some compact region, increasing the network modularity. doi:10.1371/journal.pcbi. 0020173.g002

We formed the PHN by representing the proteins as nodes and connecting two nodes if their symmetric BLAST [18] E-value was smaller than a given cutoff, *ɛ* (see Materials and Methods and Figure 2).

**Figure 3:**
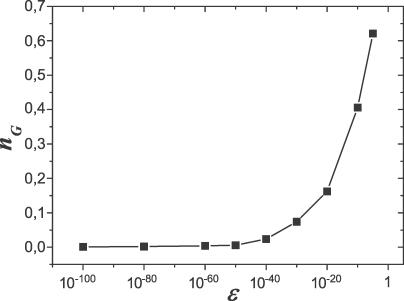
PHN Giant Component The fraction *n_G_* of nodes included in the largest connected component of the PHN is shown as a function of the homology cutoff *ɛ*. doi:10.1371/journal.pcbi. 0020173.g003

**Figure 4:**
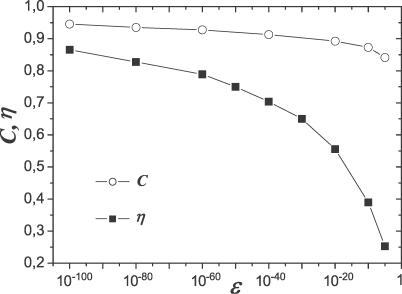
PHN Topology The compactness index, *η,* and the clustering index, *C,* shown here as a function of the E-value cutoff *ɛ,* describe the global and local topology of the network, respectively. For growing values of *ɛ, η* rapidly decreases towards 0, while *C* always has values well above 0.8. These results indicate that the PHN is formed by compact regions that are loosely connected to form globally sparse connected components. doi:10.1371/journal.pcbi. 0020173.g004

The network was formed by distinct, connected components, i.e., groups of nodes connected by a path. At the lowest values of *ɛ,* the network components were small and densely connected, including only proteins with very similar amino acid sequences. By increasing the value of *ɛ,* many of these groups of nodes merged, and the network became composed of large connected components, including proteins belonging to different families (see Figure 3). This effect is known in network science as the “emergence of the giant component,” and becomes dramatic for high values of *ɛ* when more than 60% of the proteins belong to a single connected component [19]. By measuring *η,* the index of compactness of the connected components, we see that for growing values of *ɛ* these large connected components became increasingly sparse, i.e., most pairs of nodes were not directly connected by a link (see Materials and Methods and Figure 4). At the same time, the clustering index, *C* [19], that is, a local measure of the degree of clustering of a network, varied from 0.95 to 0.84 (see Materials and Methods and Figure 4). These values of *C* are much larger than expected in the case of a random network [20], indicating a dense local structure, as found in other real world networks [19,21,22].

Taken together, these findings suggest that at the highest values of *ɛ* investigated, the PHN was formed by many compact regions, which are loosely connected by few links. We set the homology cutoff at the most permissive value, *ɛ* = 10^−5^, and we identified the compact regions of the PHN with the protein families. In this way, the problem of classifying a set of proteins into families was reduced to the problem of distinguishing the links that are internal to these compact regions from those connecting two different groups of proteins.

### Protein Families from the PHN Topology

To identify protein families, for each pair of nodes *i,j* of the PHN, we computed the overlap, θ*_ij_* (see Materials and Methods), that measures the similarity between the sets of nearest neighbors of the two nodes. Nodes within the same family have high θ*_ij_* values, while nodes belonging to different families, having few common nearest neighbors, have low θ*_ij_* values. We rewired the PHN by connecting pairs of nodes that had an overlap above a threshold value θ** (see Materials and Methods), and computed the connected components of the resulting network. For small values of θ*,* the network was still dominated by a single connected component that included a large fraction of the nodes. By increasing the cutoff θ*,* the size of the largest cluster sharply decreased, and the giant component became disconnected into a set of smaller, compact subnetworks.

**Figure 5:**
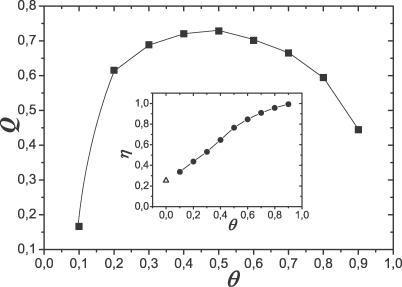
Overlap and PHN-Families By partitioning the network with the overlap procedure for increasing value of θ*,* we separated the PHN into regions of increasing compactness. The maximum value of the modularity measure *Q* (see Materials and Methods) allowed us to identify the optimal cutoff value to partition the PHN into families of homologous proteins. (Main Graph) *Q* is shown as a function of θ**. The maximum value of *Q* = 0.723 is found for θ** = 0.5. (Inset Graph) The dark circles represent the compactness index *η* after the partitioning (see Materials and Methods) as a function of θ**. The white triangle is the value of *η* of the original PHN for *ɛ* = 10^−5^, which corresponds to the limiting value θ** = 0. doi:10.1371/journal.pcbi. 0020173.g005

For different values of θ*,* we evaluated the modularity measure *Q* [23] (see Materials and Methods) of the resulting partitioning of the network. *Q* measures the extent to which a partitioning reflects the underlying community structure. After the overlap procedure on the *ɛ* = 10^−5^ network, we obtained a maximum *Q*_max_ *=* 0.723, for θ** = 0.5, as shown in Figure 5. Before applying the overlap procedure, the maximum modularity of the PHN was *Q =* 0.39 at *ɛ* = 10^−40^. The best values of *Q* observed in other systems fall in the range *Q =* 0.3 ÷ 0.7 [23,24], showing that in the PHN the modular structure is very well-defined. The maximum in *Q* for θ** = 0.5 indicates that, by linking nodes that have at least half of their nearest neighbors in common, we partitioned the network into a set of components that best coincide with the densely connected regions. In Figure 5 we also show the compactness index *η,* recalculated for different values of θ**. The value of *η* grows with θ*;* for θ** = 0.5 we obtain *η* = 0.77. This value is higher than those obtained before the overlap procedure, and confirms a strict correspondence between the connected components generated by the overlap procedure and the densely interlinked regions of the PHN.

We defined PHN-Families as the connected components when θ** = 0.5. We found 28,226 *PHN-*Families containing at least two proteins, plus 127,856 isolated proteins. The giant component of the original homology network was disconnected into 14,443 distinct PHN-Families plus 26,274 isolated proteins. Eleven percent of the connections were removed from the PHN, while the new links introduced represented about 5% of the connections.

To assess the biological relevance of the overlap procedure, we compared added and removed links with Pfam [25], a high-quality protein domain classification database (see Materials and Methods). A link added to the network by means of the overlap procedure was considered correct *iff* the two connected proteins shared at least one Pfam domain. The deletion of a link was considered correct if the two connected proteins did not belong to the same Pfam family, or at least one of them was a multidomain protein. For θ** = 0.5, 98.5% of the newly added links connected proteins that shared a classified domain, while more than three fourths of the removed links involved multidomain proteins or proteins with noncompatible classifications (see Table 1). We conclude that our unsupervised classification has a quality comparable to a classification manually curated by human experts.

**Table 1:**
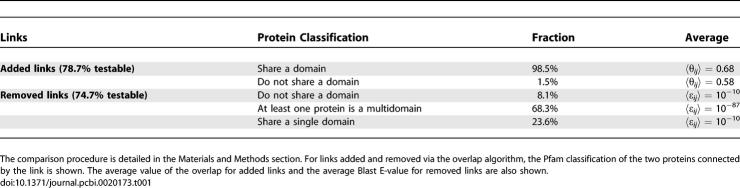
Comparison to the Pfam Database

### PHN Families in Contact-Dependent Secretion Systems

We have studied the PHN-Families containing members of selected T3SS and T4SS reference functional classes (see Protocol S1 and Table S1). Both T3SS and T4SS are characterized by a core of conserved protein classes (SctC/J/N/R/S/T/U/V for T3SS, and VirB4/6/8/9/10/11/D4 for T4SS) present in the majority of the systems, each classified in a single PHN-Family. Core proteins are accompanied by a variable number of accessory proteins belonging to less conserved functional classes, distributed in multiple PHN-Families (see Table S2, where we report the number of the corresponding PHN-Families for each functional class, and the total number of proteins included).

#### Type III secretion systems.

The conserved PHN-Families also contain proteins belonging to the related flagellar apparatus, suggesting that they represent the core machinery common to both systems. The proteins in this group are preferentially localized in the basal body (inner membrane, periplasm, and outer membrane), with the exception of SctJ, a lipoprotein whose exact localization is still unclear. We verified that all the proteins in the SctV/R/S/T/U/J PHN-Families belong either to a T3SS or to a flagellar apparatus. These PHN-Families comprise between 179 (SctJ) and 229 (SctV) proteins. The PHN-Family including the SctC proteins contains 310 members of the GspD superfamily, belonging to T3SS, flagellar apparatuses, competence systems, type II secretion system, and type IV pili. The SctN proteins are secretion-specific ATPases, and are included in a large ATPase/ATP-synthase PHN-Family with 973 members. The remaining, less conserved families are much smaller than the conserved ones, going from 25 proteins (SctK, distributed in two PHN-Families), to 181 proteins (SctQ, in three PHN-Families).

**Figure 6:**
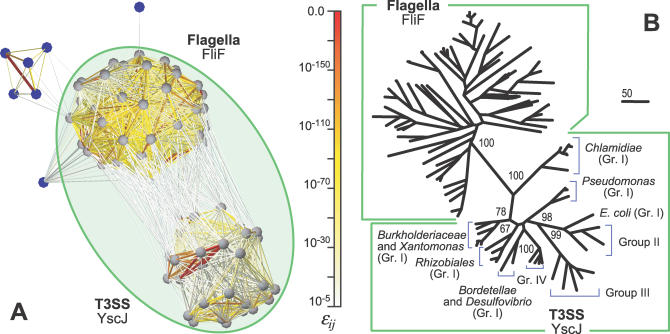
SctJ PHN-Family: Network and Phylogeny In this example we show a representation of a single PHN-family, compared with a reconstruction of the evolutionary history of its components based on molecular phylogenetic data. The two subgroups clearly visible in the PHN representation coincide with monophyletic clades of the phylogenetic tree. (A) Network representation of the SctJ PHN-family (see Protocol S1). Spheres represent proteins; edges are homology relations, color-coded according to the homology level *ɛ_ij_*. The two subgroups are YscJ (T3SS) and FliF (flagellar) proteins. For *ɛ* = 10^−5^, this portion of the PHN falls in the giant component, for the presence of false homology relations with seven outlier proteins (blue spheres, external links to the giant component not shown). After the overlap procedure with θ** = 0.5, false links are removed, and all the members of the SctJ family fall in a single PHN-family, shown by the circle. (B) Maximum likelihood phylogenetic tree of the SctJ family. Numbers are bootstrap values. The YscJ and FliF subgroups correspond to two distinct evolutionary clades. Organism and group names in the T3SS clade refer to the T3SS classification shown in Figure 8. doi:10.1371/journal.pcbi. 0020173.g006

In Figure 6 (see Figure 6A), we show a representation of the region of the PHN containing the SctJ family. Seven proteins with functional annotation incompatible with the SctJ family mediate the connection to the giant component; these outliers are not included in the SctJ family by the overlap procedure. Although all the SctJ proteins, both from T3SS and flagella, are included in a single PHN-Family, two substructures are clearly visible, corresponding to the YscJ subfamily of T3SS and to the FliF subfamily of flagellar apparatuses, respectively. In Figure 6B, a phylogenetic tree of this group of proteins is shown (see Protocol S1). The same two subgroups identified in Figure 6A form two separate, monophyletic clades of the complete tree, showing that: (i) evolutionary relationships between groups of proteins can be reliably inferred from the topology of the PHN, (ii) PHN-Families are able to recognize distant homology relationships connecting compact subgroups.

**Figure 7:**
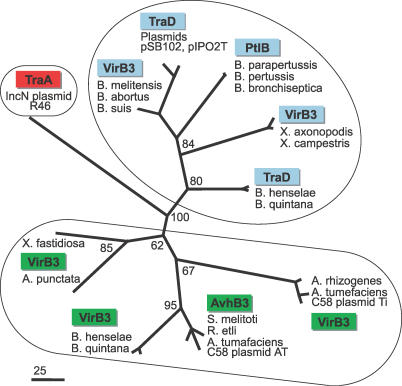
VirB3 PHN-Families Phylogeny The PHN-Families of nonconserved genes correlate with their molecular phylogeny. Shown here is the Maximum Likelihood tree of the 33 VIRB3 proteins classified in three PHN-Families (see Table S1). PHN-Families are enclosed in circles, color-coded as in Figure 8, and coincide with monophyletic branches of the phylogenetic tree. Numbers are bootstrap values, and the ruler shows the number of point-accepted mutations. doi:10.1371/journal.pcbi. 0020173.g007

**Figure 8:**
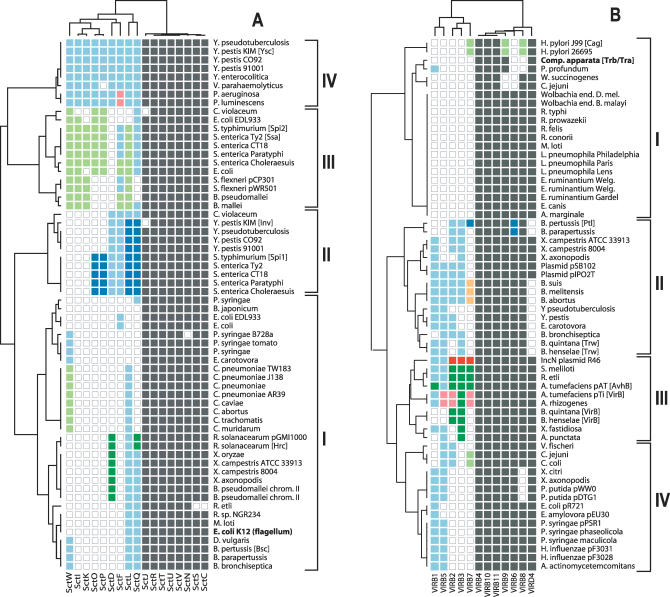
PHN-Family Based Profiling of T3SS and T4SS Multiprotein complexes can be classified by considering the presence or absence of part of their components, or their specific variants. Rows represent different T3SS (A) and T4SS (B); columns represent protein functional classes. Different colors identify different PHN-Families. Empty squares indicate absent proteins, while conserved proteins are shown in gray. Two external reference systems (E. coli flagellar apparatus and a Tra/Trb conjugative system) are marked in bold. The dendrograms represent a hierarchical clustering of the data (for further details and a list of the proteins, see Protocol S1, Table S3, and Table S4) that highlights the presence of four major groups (roman numbers) both in T3SS and T4SS. doi:10.1371/journal.pcbi. 0020173.g008

#### Type IV secretion systems.

Proteins classified in the PHN-Families associated with the VirB/D4 reference functional classes belong either to a T4SS or to a conjugative transfer apparatus. The only exceptions are the VirB11 proteins, members of a larger family of ATPases (724 proteins present in a large group of bacteria) used to energize type II and IV secretion systems, type IV pili, and competence apparatuses. The other proteins of the conserved core (VirB4/6/8/9/10/D4) belong, with minor exceptions, each to a single family containing 69 to 174 proteins. The remaining functional classes show a lower degree of conservation in different systems, and are split in two (VirB1/5), three (VirB3), four (VirB2), or six (VirB7) different PHN-Families. Proteins belonging to the conserved core are known or predicted to be involved in substrate delivery across one or both membranes, through the mating-pore–formation complex [13]. The majority of the remaining gene products contribute to the formation of the extracellular conjugative pilus, or are secreted after post-translational modifications.

For the 33 VirB3 proteins, a typical example of noncore family, the phylogenetic tree (see Protocol S1) reported in Figure 7 shows that each single PHN-Family corresponds to a monophyletic group. The same is true for the other T3SS and T4SS families (unpublished data). In the VirB3 case we also observe that the genetic distance, as measured by molecular phylogenetic analysis, can be higher between members of the same family (X. fastidiosa and Ti plasmid VirB3, 230 point-accepted mutations) than between members of different families (X. fastidiosa VirB3 and B. henselae TraD, 182 point-accepted mutations).

### Type III and Type IV Secretion Systems Profiling Based on PHN-Families

The PHN-Families composition of the reference T3SS and T4SS is a template that can be used to identify other secretory apparatuses. By looking for regions that have a similar PHN-Families composition, we identified 61 putative T3SS in 44 different genomes and 61 putative T4SS in 51 genomes, plus three broad host range plasmids (see Protocol S1, Table S3, and Table S4). A representation of these systems is shown in Figure 8. Also shown is a hierarchical clustering of the different systems (see Protocol S1) based on the PHN-Family classification of their constituents. The result is a PHN-Family–based profiling of T3SS and T4SS that allows us to distinguish different groups of secretory apparatuses.

#### Type III secretions systems.

Four groups of T3SS [26–28], indicated by the roman numbers I–IV, with several subgroupings, can be identified in Figure 8A: group I shows a composite group that includes the ancestrally related flagellar export machinery in E. coli K12; group II is the *Salmonella* SPI-2 system; group III is the *Salmonella* SPI-1 system; and group IV is the *Yersinia* Ysc system of the pCD1 plasmid. Due to the lack of most of the proteins characterizing the T3SS, group I appears to have evolved early after the divergence of T3SS from the flagellar export apparatuses. The systems in groups II, III, and IV probably formed later, as confirmed by the molecular phylogenetic analysis on SctJ conserved genes, shown in Figure 6B, by the recruitment of a variable number of specialized proteins. It is also evident from Figure 8A that, while the proteins specific to group IV could have been acquired in a single event, at least two independent horizontal transfer events are required for the formation of systems in both group II and group III.

#### Type IV secretion systems.

We identified four groups of T4SS, as shown in Figure 8B. Group I includes 33 Tra/Trb identical conjugative apparatuses (only one representative is shown in Figure 8B) and the H. pylori Cag apparatus, whose VirB7/8/9 genes have differentiated so much from their ancestors that they are no longer classified in the respective core families. Group II is characterized by the VirB1/2/3/5 proteins of the pSB102/pIPO2T broad host range plasmids; group III by the VirB3 (and to a minor extent VirB2/7) proteins of the A. tumefaciens VirB apparatus; organelles in group IV complement the core set with only one or two accessory proteins (VirB1/5) shared with both the A. tumefaciens VirB and the pSB102/pIPO2T operon. In group IV we also found C. jejuni and C. coli plasmids, whose VirB7 proteins belong to the same small family as the H. pylori Cag (group I) homologues. This incongruence, along with the small VirB6 family of the *Bordetellae* Ptl system and the nonhomogeneous pattern of the VirB1/2/3/5/7 PHN-Families in *Agrobacterii, Rhizobii, Bartonellae,* and *Xylellae* of group III, again suggest that distinct genetic units have been recruited independently to complement the core proteins.

## Discussion

We have generated a network formed by the homology relationships amongst proteins, as inferred by primary sequence conservation. The regions of aggregation of the network correspond to protein families, whose members are evolved from a common ancestor with different degrees of diversification. We have partitioned the network with an algorithm that identifies the dense regions and allows the definition of the PHN-Families of homologous proteins. The method does not require human intervention and is based solely on local properties of the network. A comparison with an external protein domain database suggests that this approach produces results with a quality comparable to the ones generated by human experts.

To demonstrate the potential of this approach, we have selected bacterial organelles consisting of large numbers of interacting proteins, namely T3SS and T4SS, in which individual components require high reciprocal specificity to perform their functions. The PHN-Families were found to provide a coherent and comprehensive classification of secretons. A comparison of the PHN-Family classification of proteins performing specific functions with a molecular phylogenetic analysis of the same proteins suggests that the PHN-Families are consistent with the evolutionary patterns even when family members have undergone sharp, asymmetric genetic divergences.

We have identified 61 T3SS and 61 T4SS in our dataset, and we have compared them using a PHN-Family based profiling. We found that they can be classified into groups that are consistent with the molecular phylogeny of the conserved proteins. Nevertheless, some of the noncore functional classes show a distribution across the hierarchical groups that are not compatible with the main evolutionary path, suggesting that the secretory apparatuses were not acquired in single events.

Rather, the results suggests that, for both T3SS and T4SS, a conserved module remained substantially unmodified since the ancestral duplication that led to the diversification of T3SS and T4SS from flagellar and conjugative apparatuses, respectively, and it has been complemented during evolution with distinct genetic units, recruited independently, and adapted to build a variety of specialized contact-dependent secretion systems. This process appears to occur in discrete steps, in which a system progressively adapts to novel substrates by the exchange of relatively large amounts of genetic information with other organisms.

In summary, the PHN-Families provide a comprehensive catalogue of the protein repertoire, also useful for the detection of inheritance patterns. The results obtained on the diversification of the Type III and IV secretory apparatuses open the possibility to conduct a detailed study on the evolutionary events that led to their formation, where different hypotheses could be formulated and thoroughly tested. Furthermore, an extension of this analysis beyond the structural components of secretory apparatuses could allow us to characterize more elusive elements, such as previously unknown effectors and regulators.

Given the increasing number of bacterial genome sequences, and the number of genes with unknown function [29,30], PHN-Families could provide a powerful annotation tool, allowing straightforward comparisons of whole genomes and the discovery of novel and previously uncharacterized functions.

## Materials and Methods

### Sequence dataset.

The amino acidic sequences of 761,260 proteins from 251 completely sequenced bacterial genomes and 716 bacterial plasmids were downloaded from the National Center for Biotechnology Information Web site, http://www.ncbi.nlm.nih.gov, (see Table S5). An all-against-all Blast [18] search was performed, and a matrix containing the Blast E-value was obtained. The search was performed using blastp version 2.2.11 with the BLOSUM62 substitution matrix, filtering the low complexity regions and not using composition-based statistics. Remaining search parameters were left to the default values.

Since the E-value is not invariant for the exchange of the query and target sequences, we define the symmetric E-value *ɛ_i,j_* between the proteins *i,j* as:

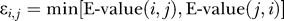



With this definition, the alignment between each pair of sequences is weighted by its most favorable E-value. Different from the reciprocal-best-hit method, frequently used to identify ortholog proteins in different genomes, proteins are allowed to have multiple hits in a single genome. Thus, the PHN includes links between paralogs that would be discarded using the reciprocal-best-hit method, giving a more complete picture of the PHN topology.

### PHN topological quantities.

We defined the PHN as the network where the proteins are the nodes, and two nodes, *i,j,* are connected by an undirected edge *iff* ɛ*_i,j_* is smaller than a given threshold *ɛ*. While the number of vertexes, *N,* in the graph (network size) is fixed by the number of proteins in the dataset, the number of links, and consequently the structure of the network, depends on the cutoff adopted. For *ɛ* = 10^−180^, 1.0 × 10^6^ links are present. With increasing values of *ɛ,* more links are included in the network, causing the connected components to merge. For *ɛ* = 10^−5^, the highest value of *ɛ* considered, the network contains above 6.6 × 10^7^ links.

We partitioned the PHN with a single-linkage clustering algorithm. Two nodes are in the same connected components if there is a path connecting them. For *ɛ* = 10^−180^, we found 6.4 × 10^5^ connected components, and 84% of the nodes in the network were singlets, *i.e.,* isolated nodes. The number of connected components decreased with increasing *ɛ*. For *ɛ* = 10^−5^ we found 8.9 × 10^4^ connected components, and only 8% of the nodes were singlets, while the largest connected component contained more than 60% of the whole PHN (see Figure 3).

*Compactness index.* For a given partitioning of the network, we define:

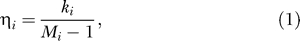
where *k_i_* is the number of links departing from the *i*-th node, *M_i_* is the number of nodes in the same partition, and *η_i_* represents the fraction of nodes in the same partition as the node *i* that are also nearest neighbors of *i*. The compactness index, *η,* is the average of *η_i_* over the *N* non-isolated nodes of the network:

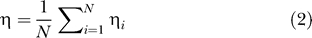



Isolated nodes are excluded from the average. *η* coincides with the average, over all the connected components, of the fraction of links compared with a clique of the same size, where each connected component is weighted by its size.

In a clique, all nodes are nearest neighbours, and therefore all have *η_i_ =* 1, while *η_i_ ≈* 0 if a connected component is sparse. For *ɛ* = 10^−100^, more than 70% of the proteins in the PHN have *η_i_* very close to 1: the network is dominated by connected components that are very close to cliques. This fraction decreases to less than 20% for *ɛ* = 10^−5^, showing that the network becomes increasingly sparse.

***Clustering index***. The local degree of compactness of a network is measured by the clustering index, *C_i,,_* and by its average over the entire network, *C*. The clustering index of a node *i* is defined [19] as:

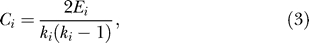
where *E_i_* is the number of edges among the *k_i_* nearest neighbors of *i*. The average network clustering index, *C,* is given by:

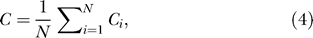
where *N* is the number of nodes in the network. *C_i_* is 1 for a node at the centre of a fully interlinked region, i.e., if all its nearest neighbours are also directly connected, and is 0 for a protein at the centre of a star topology. As shown in Figure 4, the network is always dominated by nodes with high clustering index. *C* decreases only from 0.95 for *ɛ* = 10^−180^ to 0.84 for *ɛ* = 10^−5^, and the shape of the distribution of *C_i_* is only slightly dependent on *ɛ,* indicating that the PHN local topology is substantially independent on the evolutionary distance considered in protein homology relations. In a homogeneous random network [20] of the same size, the clustering index, *C*_rand_, would vary from *C*_rand_ = 1.7 × 10^−6^ to *C*_rand_ = 1.1 × 10^−4^.

*Overlap*. We define the overlap θ*_ij_* of two nodes, *i,j* as:

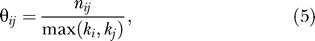
where *n_ij_* is the number of nearest neighbors common to node *i* and node *j*, and *k_i_* and *k_j_* are the number of nearest neighbors of node *i* and *j,* respectively. For a similar topological quantity, see [21].

If two nodes belong to a clique, their overlap θ*_ij_* is 1, and, in general, two nodes belonging to the same densely connected region have a value of θ*_ij_* close to 1; nodes belonging to different communities have little overlap.

Network rewiring. To identify the densely connected regions of the network, for each pair of nodes *i,j* we calculate their overlap θ*_ij_* using the PHN at *ɛ* = 10^−5^. Then we rewire the PHN connecting two proteins *iff* their overlap θ*_ij_* is larger than a given cutoff 0 < θ** ≤ 1. With this procedure, only links connecting nodes that share a certain degree of similarity between their nearest neighbor shells are retained. Nodes belonging to different communities are disconnected, while new links between nodes that were only second-nearest neighbors in the original network are introduced. Consequently, each value of θ** corresponds to a set of connected components, that we use as a partitioning of the PHN at *ɛ* = 10^−5^.

Modularity measure. The extent to which a network partitioning captures the underlying community structure is quantified by the modularity measure, *Q* [23]. For a given partitioning of the network, *Q* is defined [23] as:

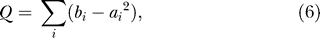
where *a_i_* is the fraction of edges with at least one end in the *i*-th component, and *b_i_* is the fraction of edges with both ends in the *i*-th component. *Q* measures the correlation between the topology of the network and its partitioning. For a randomly partitioned network, *Q* = 0. If a partitioning corresponds to the communities present in the network, its modularity approaches the maximum.

### Comparison with the Pfam database.

Pfam [25] is a curated collection of multiple alignments of protein domains or conserved protein regions (http://pfam.wustl.edu/). Pfam version 12.0 was used, including 7,316 families in Pfam-A and 108,951 in Pfam-B. Proteins are classified in a Pfam family if they own a specific domain. Unlike the PHN-Families, the same protein can be classified in more than one Pfam family, since a protein can include more than one domain. Only 78.7% of the new links introduced and 74.7% of the links removed by the overlap procedure in the PHN connected proteins annotated in Pfam, and were evaluated.

Results are shown in Table 1; 98.5% of the added links connect proteins sharing at least one domain, confirming the ability of this method to identify distant homologies. Also shown are the average overlap values for the added links. A lower value was observed for the small fraction of links connecting proteins that do not share an annotated Pfam domain.

Eight and one tenth percent of the removed links connect proteins not sharing a Pfam domain, and 68.3% connect at least one multidomain protein. Since our procedure does not allow us to classify a protein in more than one family, we consider the deletion of these links as correct. Taken together, these two cases include 76.4% of the removed links. In the remaining 23.6% of the cases, the removed links connect proteins sharing a single domain in Pfam, and therefore the removal of these links are considered incorrect, although the possibility exists that these proteins include domains not yet classified by Pfam. Also shown in Table 1 are the average E-values of the removed links. Links involving multidomain proteins are characterized by a much stronger homology than the other removed links.

## Supporting Information

Protocol S1 Supplementary Methods (48 KB DOC)

Table S1 T3SS and T4SS Reference Dataset Each column is a secretory apparatus, each row a functional class; in each cell, protein name and protein GI number are shown. T3SS: *Pseudomonas aeruginosa, Ralstonia solanacearum, Salmonella typhimurium, Xanthomonas campestris,* and Yersinia pestis. Functional classes according to [7]. T4SS: Agrobacterium tumefaciens (VirB/D4 and AvhB operons), *IncN plasmid R46* (Tra operon), Brucella suis (VirB operon), Bordetella pertussis (Ptl operon), and Helicobacter pylori (Cag operon). Functional classes using the A. tumefacines VirB operon as a prototype.
(133 KB DOC)

Table S2 T3SS and T4SS PHN-Families For each component of T3SS (A) and T4SS (B), the number of PHN-Families and their size (number of proteins per family) are shown.
(52 KB DOC)

Table S3 Proteins Included in the Identified T3SS Detailed list of T3SS proteins shown in Figure 8A.
(74 KB XLS)

Table S4 Proteins Included in the Identified T4SS Detailed list of T4SS proteins shown in Figure 8B.
(53 KB XLS)

Table S5 Protein Dataset Complete genomes and plasmids from which proteins where predicted.
(101 KB XLS)

## Abbreviations

Pfam: protein family database
PHN: protein homology network
PHN-Families: families of homologous proteins identified with the PHN method
T3SS: Type III secretion systems
T4SS: Type IV secretion systems
