# Supplementary material for: Protein Homology Network Families Reveal Step-Wise Diversification of Type III and Type IV Secretion Systems
Source: PLoS Comput Biol. 2006 Dec 1;2(12):e173. doi: 10.1371/journal.pcbi.0020173 (PMC1676029; doi:10.1371/journal.pcbi.0020173)
Supplement: Supplementary file 1 [file pcbi.0020173.sd001.doc]

### Supplementary Methods

### PHN visualizations

Two small portions of the PHN – the nearest neighbours of the VirB1 family at *ε* = 10^-5^ in Fig.1 and a small fraction of the giant component (39.321 nodes, 4.4 x10^6^ links at *ε* = 10^-30^) in Fig.2 – are visualized by using the bi-dimensional version of the lgl-layout algorithm in the LGL suite [1], with an ad-hoc postscript driver written by the authors. The SctJ-associated portion of the PHN is visualized in Fig.6 with the aid of the Tulip 2.0.0 graphic library (<http://www.labri.fr/perso/auber/projects/tulip/>) by using the 3Gem algorithm.

### Molecular phylogenetic analyses

All the molecular phylogenetic investigations have been performed by:

- multiple alignment of proteins included in the single PHN-family under investigation (core functional classes) or included in all the PHN-familes associated with the non-core functional class, with clustalw1.83[2];
- 100 replicates bootstrap of the sequence alignment with SEQBOOT[3];
- for each replicate, maximum likelihood phylogeny with PROML[3];
- consensus tree with CONSENSE[3], using the majority rule extended;
- for the original multiple alignment, maximum likelihood phylogeny with PROML[3], constrained by the consensus tree topology;
- graphical output with TreeView 1.6.6 (<http://taxonomy.zoology.gla.ac.uk/rod/rod.html> )

### Secretion systems reference functional classes.

To constitute the reference functional classes for T3SS and T4SS, we have identified the major structural components of 7 T3SSs from 5 bacteria, and 6 T4SSs from 4 bacteria and a broad host range plasmid (see Table	S1). T3SS proteins have been classified in seventeen functional groups (SctC/D/F/I-L/N/W) according to the unified nomenclature proposed in [4]. T4SS proteins have been classified in twelve functional groups (VirB1-11/D4) using the *A. tumefaciens* VirB operon as a prototype[5].

### Identification of Type III and Type IV secretion systems.

We have identified T3SSs by requiring that a DNA molecule contains at least one member of 5 of the conserved families common both to T3SS and to flagella (SctC, SctJ, SctN, SctR, SctS, SctT, SctU, SctV). Similarly, we have identified T4SSs by requiring that a DNA molecule contains at least one member of 5 of the conserved familiesVirB4/6/8/9/10/11/D4.

In order to distinguish T3SSs/T4SSs from flagella/conjugative apparatuses we performed a preliminary, automated screening, followed by a manual investigation based on genome annotation, gene localization on the chromosomes and protein sequence.

To call a T3SSs we required the presence of at least a member of one of the families specific to T3SSs (SctD, SctF, SctI, SctK, SctL, SctO, SctP, SctQ). To call a T4SSs the presence of a VirB6 or of a non-core protein (VirB1/2/3/5/7) was required.

The successive manual investigation lead to the inclusion of the *B.japonicum* Rhc secretion apparatus (Accession:BA000040 in which only the core proteins rhcC2/J/N/R/S/T/U/V proteins are represented), and to the inclusion of two T4SSs (*Wolinella succinogenes*, Accession:BX571656, and the *Campylobacter jejuni* plasmid pVir, Accession: NC_005012).

Tables S3 and S4 report the complete list of all the proteins included in the identified secretory apparatuses, respectively for T3SS and T4SS.

### Hierarchical clustering of T3SS and T4SS

Each secretion system is mapped upon a list of family names, with empty positions if no representative of a specific family are found in a secretion system. We compute a distance matrix between all pairs of systems by using the routine *daisy* from the statistical package *cluster* included in the program R version 2.0.0. The distance between two systems is given by the fraction of positions occupied by proteins belonging to different PHN-families. The clustering is an agglomerative hierarchical clustering performed by using the routine *agnes* from the *cluster* package in the R program with the Ward method[6].

### Bibliography

1. Adai AT, Date SV, Wieland S, Marcotte EM (2004) LGL: creating a map of protein function with an algorithm for visualizing very large biological networks. J Mol Biol 340: 179-190.

2. Thompson JD, Higgins DG, Gibson TJ (1994) CLUSTAL W: improving the sensitivity of progressive multiple sequence alignment through sequence weighting, position-specific gap penalties and weight matrix choice. Nucleic Acids Res 22: 4673-4680.

3. Felsenstein J (2005) PHYLIP (Phylogeny Inference Package) version 36 Distributed by the author Department of Genome Sciences, University of Washington, Seattle.

4. Hueck CJ (1998) Type III protein secretion systems in bacterial pathogens of animals and plants. Microbiol Mol Biol Rev 62: 379-433.

5. Covacci A, Telford JL, Del Giudice G, Parsonnet J, Rappuoli R (1999) Helicobacter pylori virulence and genetic geography. Science 284: 1328-1333.

6. Kaufman L, Rousseeuw PJ (1990) Finding Groups in Data. An Introduction to Cluster Analysis. New York: Wiley.
