# Supplementary material for: Protein Homology Network Families Reveal Step-Wise Diversification of Type III and Type IV Secretion Systems
Source: PLoS Comput Biol. 2006 Dec 1;2(12):e173. doi: 10.1371/journal.pcbi.0020173 (PMC1676029; doi:10.1371/journal.pcbi.0020173)
Supplement: Supplementary file 2 [file pcbi.0020173.st001.doc]

|  | **Pseudomonas aeruginosa** | **Ralstonia solanacearum** | **Salmonella typhimurium LT2 SPI-1** | **Salmonella typhimurium LT2 SPI-2** | **Xanthomonas campestris** | **Yersinia pestis chromosome** | **Yersinia pestis plasmid** |
| --- | --- | --- | --- | --- | --- | --- | --- |
| **SctV** | PcrD | HrcV | InvA | SsaV | HrcV | Ypo0266 | LcrD |
|  | 9947677 | 17431335 | 16421444 | 16419935 | 21112275 | 15978366 | 5832454 |
| **SctW** | PopN |  | InvE |  |  |  | YopN |
|  | 9947672 |  | 16421445 |  |  |  | 5832459 |
| **SctN** | PscN | HrcN | InvC | SsaN | HrcN | Ypo0267 | YscN |
|  | 9947670 | 17431342 | 16421442 | 16419936 | 21112282 | 15978367 | 5832460 |
| **SctO** | PscO |  | InvI | SsaO |  |  | YscO |
|  | 9947669 |  | 16421441 | 16419937 |  |  | 5832461 |
| **SctP** |  |  | InvJ | SsaP |  |  | YscP |
|  |  |  | 16421440 | 16419938 |  |  | 5832462 |
| **SctQ** | PscQ | HrcQ | SpaO | SsaQ | HrcQ | Ypo0269 | YscQ |
|  | 9947667 | 17431333 | 16421439 | 16419939 | 21112272 | 15978370 | 5832463 |
| **SctR** | PscR | HrcR | SpaP | SsaR | HrcR | Ypo0270 | YscR |
|  | 9947666 | 17431332 | 16421438 | 16419940 | 21112271 | 15978371 | 5832464 |
| **SctS** |  | HrcS | SpaQ | SsaS | HrcS | Ypo0271 | YscS |
|  |  | 17431331 | 16421437 | 16419941 | 21112270 | 15978372 | 5832465 |
| **SctT** | PscT | HrcT | SpaR | SsaT | HrcT | Ypo0272 | YscT |
|  | 9947664 | 17431344 | 16421436 | 16419942 | 21112284 | 15978373 | 5832466 |
| **SctU** | PscU | HrcU | SpaS | SsaU | HrcU | Ypo0273 | YscU |
|  | 9947663 | 17431336 | 16421435 | 16419943 | 21112276 | 15978374 | 5832467 |
| **SctC** | PscC | HrcC | InvG | SsaC | HrcC | Ypo0257 | YscC |
|  | 9947691 | 17431346 | 16421446 | 16419915 | 21112285 | 15978356 | 5832472 |
| **SctD** | PscD | HrpW |  | SsaD | HrpD5 | Ypo0258 | YscD |
|  | 9947692 | 17431329 |  | 16419916 | 21112268 | 15978357 | 5832473 |
| **SctF** | PscF |  | PrgI |  |  | Ypo0260a | YscF |
|  | 9947694 |  | 16421420 |  |  | 15978360 | 5832475 |
| **SctI** | PscI |  | PrgJ |  |  |  | YscI |
|  | 9947697 |  | 16421419 |  |  |  | 5832478 |
| **SctJ** | PscJ | HrcJ | PrgK | SsaJ | HrcJ | Ypo0263 | YscJ |
|  | 9947698 | 17431339 | 16421418 | 16419930 | 21112279 | 15978363 | 5832479 |
| **SctK** | PscK |  | OrgA |  |  |  | YscK |
|  | 9947699 |  | 16421416 |  |  |  | 5832480 |
| **SctL** | PscL | HrpF | OrgB | SsaK | HrpB5 | Ypo0265 | YscL |
|  | 9947700 | 17431341 | 16421417 | 16419932 | 21112281 | 15978365 | 5832481 |

**T3SS**

|  | **Agrobacterium tumefaciens VirB (Ti plas.)** | **Agrobacterium tumefaciens AvhB (At plas.)** | **IncN plasmid** | **Brucella suis** | **Bordetella pertussis** | **Helicobacter pylori** |
| --- | --- | --- | --- | --- | --- | --- |
| **VirB1** | VirB1 | avhB1 | TraL | VirB1 |  |  |
|  | 17744197 | 17743593 | 17530588 | 23463406 |  |  |
| **VirB2** | VirB2 | avhB2 | TraM | VirB2 | PtlA |  |
|  | 17744198 | 17743594 | 17530590 | 23463404 | 33564719 |  |
| **VirB3** | VirB3 | avhB3 | TraA | VirB3 | PtlB |  |
|  | 17744199 | 17743595 | 17530591 | 23463403 | 33564720 |  |
| **VirB4** | VirB4 | avhB4 | TraB | VirB4 | PtlC | CagE |
|  | 17744200 | 17743596 | 17530592 | 23463402 | 33564721 | 2313659 |
| **VirB5** | VirB5 | avhB5 | TraC | VirB5 |  |  |
|  | 17744201 | 17743597 | 17530593 | 23463401 |  |  |
| **VirB6** | VirB6 | avhB6 | TraD | VirB6 | PtlD |  |
|  | 17744203 | 17743599 | 17530595 | 23463400 | 33564722 |  |
| **VirB7** | VirB7 | avhB7 | TraN | VirB7 | PtlI | CagT |
|  | 17744204 | 17743600 | 17530596 | 23463399 | 33564723 | 2313648 |
| **VirB8** | VirB8 | avhB8 | TraE | VirB8 | PtlE | HP530 |
|  | 17744205 | 17743601 | 17530597 | 23463398 | 33564724 | 2313645 |
| **VirB9** | VirB9 | avhB9 | TraO | VirB9 | PtlF | Cag8 |
|  | 17744206 | 17743602 | 17530598 | 23463397 | 33564725 | 2313643 |
| **VirB10** | VirB10 | avhB10 | TraF | VirB10 | PtlG | Cag7 |
|  | 17744207 | 17743603 | 17530599 | 23463396 | 33564726 | 2313642 |
| **VirB11** | VirB11 | avhB11 | TraG | VirB11 | PtlH | Cagα |
|  | 17744208 | 17743604 | 17530600 | 23463395 | 33564727 | 2313639 |
| **VirD4** | VirD4 |  | TraJ |  |  | Cag5 |
|  | 17744216 |  | 17530604 |  |  | 2313638 |

**T4SS**

**Table S1. T3SS and T4SS reference dataset.** Each column is a secretory apparatus, each row a functional class, in each cell protein name and protein GI number are shown. **T3SS**: *Pseudomonas aeruginosa*, *Ralstonia* *solanacearum*, *Salmonella* *typhimurium*, *Xanthomonas* *campestris* and *Yersinia* *pestis*. Functional classes according [7]. **T4SS**: *Agrobacterium tumefaciens* (VirB/D4 and AvhB operons), *IncN plasmid R46* (Tra operon), *Brucella suis* (VirB operon), *Bordetella pertussis* (Ptl operon) and *Helicobacter pylori* (Cag operon). Functional classes using the *A. tumefacines* VirB operon as a prototype.
