# Supplementary material for: Protein Homology Network Families Reveal Step-Wise Diversification of Type III and Type IV Secretion Systems
Source: PLoS Comput Biol. 2006 Dec 1;2(12):e173. doi: 10.1371/journal.pcbi.0020173 (PMC1676029; doi:10.1371/journal.pcbi.0020173)
Supplement: Supplementary file 3 [file pcbi.0020173.st002.doc]

| **Functional Class** | **Families** | **Proteins** | | | |
| --- | --- | --- | --- | --- | --- |
| SctV | 1 | 193 | | | |
| *SctW* | 2 | 21 | | 23 | |
| *SctN* | 1 | 973 | | | |
| *SctO* | 3 | 5 | 8 | | 14 |
| *SctP* | 3 | 8 | 5 | | 13 |
| *SctQ* | 3 | 9 | 2 | | 170 |
| *SctR* | 1 | 185 | | | |
| *SctS* | 1 | 183 | | | |
| *SctT* | 1 | 181 | | | |
| *SctU* | 1 | 229 | | | |
| *SctC* | 1 | 310 | | | |
| *SctD* | 2 | 25 | | 8 | |
| *SctF* | 3 | 14 | 12 | | 12 |
| *SctI* | 2 | 13 | | 14 | |
| *SctJ* | 1 | 179 | | | |
| *SctK* | 2 | 11 | | 14 | |
| *SctL* | 3 | 9 | 115 | | 12 |

**A**

| **Functional Class** | **Families** | **Proteins** | | | | | | | | |
| --- | --- | --- | --- | --- | --- | --- | --- | --- | --- | --- |
| *VirB1* | 2 | 42 | | | | | 4 | | | |
| *VirB2* | 4 | 18 | | 9 | | | 7 | | 1 | |
| *VirB3* | 3 | 19 | | | 13 | | | 1 | | |
| *VirB4* | 1 | 228 | | | | | | | | |
| *VirB5* | 2 | 46 | | | | | 7 | | | |
| *VirB6* | 2 | 117 | | | | 3 | | | | |
| *VirB7* | 6 | 7 | 7 | | 5 | | 3 | 1 | | 1 |
| *VirB8* | 2 | 69 | | | | | 2 | | | |
| *VirB9* | 2 | 127 | | | | | 2 | | | |
| *VirB10* | 1 | 119 | | | | | | | | |
| *VirB11* | 1 | 724 | | | | | | | | |
| *VirD4* | 1 | 174 | | | | | 5 | | | |

**B**

**Table S2: T3SS and T4SS PHN-Families.** For each component of T3SSs (**A**) and T4SSs (**B**), the number of PHN-families and their size (number of proteins per family) are shown.
